# Cognitive Abilities and Executive Functions as Predictors of Adaptive Behavior in Preschoolers with Autism Spectrum Disorder and Typically Developing Children: A Comparative Study

**DOI:** 10.1007/s10802-025-01341-x

**Published:** 2025-06-18

**Authors:** Barbara Rašková, Margaréta Hapčová, Hana Celušáková, Daniela Franková, Mária Kopčíková, Diana Demkaninová, Jakub Januška, Katarína Babinská

**Affiliations:** 1 Institute of Physiology, Faculty of Medicine, Comenius University in Bratislava, Bratislava, 811 08 Sasinkova 2 Slovak Republic; 2 Department of Psychology, Faculty of Arts, Comenius University in Bratislava, Bratislava, Slovak Republic; 3 Psychiatric Clinic, Faculty of Medicine, Slovak Medical University, Bratislava, Slovak Republic; 4 Department of Pediatric Psychiatry, Faculty of Medicine, Comenius University in Bratislava, Child University Hospital, Bratislava, Slovak Republic

**Keywords:** Autism spectrum disorder, Executive functions, Adaptive behavior, Cognitive abilities, Preschool children

## Abstract

Children with Autism Spectrum Disorder (ASD) differ in adaptive functioning, executive functioning, and the distribution of intellectual abilities compared to their typically developing (TD) peers. Research consistently demonstrates a link between executive functions and various aspects of adaptive behavior. This study aims to compare cognitive abilities, executive functions, and adaptive behavior in non- or minimally verbal preschool-aged (3–6 years) children with ASD (*n* = 53) and TD peers (*n* = 79). The study examines broad cognitive abilities and executive functions as predictors of adaptive behavior. For this purpose, the Vineland Adaptive Behavior Scales, Third Edition, the Behavior Rating Inventory of Executive Functioning–Preschool Version, and the SON-R 2½-7 intelligence test were utilized. The results indicate that non- or minimally verbal preschoolers with ASD exhibit significant executive difficulties, particularly in working memory, and show marked differences in cognitive abilities compared to TD controls. Significant group differences were observed across all adaptive behavior domains, with the largest effect on *Socialization* and the smallest on Daily Living Skills. In the ASD group, executive functions and cognitive domains accounted for a substantial portion of the variance in the *Communication* domain, with slightly less influence observed in Daily Living Skills and Socialization. In contrast, the significant role of cognitive abilities and executive difficulties as predictors of adaptive behavior in everyday functioning was not confirmed for TD children. These findings highlight the critical role of executive functioning as a predictor of adaptive behavior in preschool children with ASD.

**Supplementary Information:**

The online version contains supplementary material available at 10.1007/s10802-025-01341-x.

## Introduction

### Background and Context

Autism spectrum disorder (ASD) is a neurodevelopmental disorder defined by difficulties in initiating and sustaining reciprocal social interactions and effective social communication. It is also characterized by restricted, repetitive, and inflexible patterns of behavior and interests or atypical activities, with varying levels of intellectual and language abilities (World Health Organization, 2019). The core challenges related to social interaction, communication, and repetitive behaviors in ASD usually become noticeable within the first two years of life (Estes et al., 2015a, b).

Adaptive behavior (AB) refers to skills that help a person manage their daily life without needing someone else’s assistance (Sparrow et al., 2016). These skills are crucial for managing tasks at home, in the community, and for personal care (Peterson et al., 2015). AB includes conceptual, social, and practical skills: conceptual skills involve reading, writing, problem-solving, and decision-making; social skills pertain to interpersonal interactions, relationships, social responsibility, and rule-following; and practical skills cover self-care, health and safety, occupational abilities, money management, and mobility (WHO, 2022).

The level of adaptive functioning varies considerably in children with ASD (Alvares et al., 2020; Bradshaw et al., 2019; Pathak et al., 2019). Children with ASD, including those with or without intellect deficits, score lower on AB tests than TD peers, with the largest gaps in social skills (Alvares et al., 2020; McLean et al., 2014; Pugliese et al., 2015; Terroux et al., 2024).

The distribution of intellectual abilities in children with ASD is different from the general population. According to data from the USA from 2020, 37.9% of children with ASD met the criteria for intellectual disability (IQ < 70), 23.5% were in the borderline range (i.e., IQ 85 − 71), and 38.6% of the children had normal intellect (IQ > 85). In children with ASD, intellectual disabilities were more commonly identified in girls (42.1%) than in boys (36.9%) (Maenner, 2023). Cognitive abilities in children with ASD often show more marked variability compared to the general population (Celušáková et al., 2021; Takayanagi et al., 2022), with primarily intact local information processing abilities and difficulties with processing speed and working memory in school age. However, findings in preschool children with ASD are less conclusive due to the limited number of studies. Some studies reported dominance in visuospatial and nonverbal abilities (Coolican et al., 2008; Kuschner et al., 2007; Nowell et al., 2015). Impairments in low processing speed and verbal abilities have been found (Hedvall et al., 2013).

Individuals with ASD and an IQ < 70 were often labeled as “low-functioning”, while those with an IQ > 70 were labeled as “high-functioning”, although this distinction had no support in any valid diagnostic classification. The AB level does not correspond with cognitive abilities, particularly in ASD individuals without intellectual disability, where AB ratings are one to two standard deviations below the population mean (Alvares et al., 2020; Hyo Jung Lee & Hye Ran Park,^ 2007^; Pathak et al., 2019). Research suggests that a discrepancy between cognitive and adaptive skill levels emerges in children with ASD as early as the age of 3 years (Bradshaw et al., 2019; Salomone et al., 2018), and this discrepancy increases later in childhood and adolescence (Alvares et al., 2020; Pathak et al., 2019). Executive functions (EFs) are higher cognitive functions that manage and control various systems, processes, and capabilities (Goldstein & Naglieri 2014). EFs are crucial for intentional reasoning, deliberate actions, emotional regulation, complex social functioning, self-regulation, and adaptation to changing conditions (Cristofori et al., 2019; Diamond, 2013; Zelazo, 2015). A number of studies support a three-factor model of EFs that includes working memory, inhibition, and cognitive flexibility (Diamond, 2013). Research in children has yielded mixed results about the structure of EFs. Some studies with preschoolers support a single general factor model (Brydges et al., 2014; Laureys et al., 2022; Xu et al., 2013; Wiebe et al., 2011), while other studies report a two-factor solution, where one factor is working memory and the other is inhibition (Monette et al., 2015; van der Ven et al., 2013). Shifting or cognitive flexibility has been associated with working memory in some studies (Monette et al., 2015) and with inhibition in others (van der Ven et al., 2013). Evidence for a three-factor model emerges at the end of preschool age (Garon et al., 2014; Schirmbeck et al., 2022) and later.

Intelligence is a multifaceted construct that includes broad and narrow cognitive abilities such as visuospatial and language abilities, reasoning, learning, etc. (Schneider & McGrew, 2012). Intelligence and EFs are closely related but are separate constructs with certain neural correlates (Barbey et al., 2012; Engelhardt et al., 2016). In the preschool period, this relationship is stronger (Nelson et al., 2016). The overlap between EFs and intellect is mainly in fluid abilities such as reasoning (Grobe et al., 2024). An open question is whether a specific component of EF uniquely predicts a particular cognitive ability (Garon et al., 2014).

EFs are associated with communication and language skills (Kaushanskaya et al., 2017; Lohndorf et al., 2019; Lonigan et al., 2017), academic skills (Butterfuss & Kendeou, 2018; Cragg et al., 2017) and social and behavioral competence (Benavides-Nieto et al., 2017; Demkaninová et al., 2022; Jacobson et al., 2011; Panerai et al., 2014). Specifically, inhibition can predict cooperative and non-cooperative behavior (Ciairano et al., 2007).

Children with ASD have significant impairments in EFs compared to their typically developing (TD) peers (Demetriou et al., 2018; Lai et al., 2017; Zhang et al., 2020), particularly in the areas of shifting (Kimhi et al., 2014; Smithson et al., 2013), inhibition (Garon et al., 2018; Valeri et al., 2020), working memory (McClain et al., 2022) and planning (Smithson et al., 2013; Verté et al., 2006). Effect sizes reported in studies range from medium to large. The broad executive dysfunction in ASD is relatively stable across development (Demetriou et al., 2018).

Research consistently demonstrates a significant relationship between EFs and AB in children with ASD. Multiple studies have found that executive skills, particularly working memory, inhibition, and self-monitoring, predict AB across various domains, including communication, socialization, and daily living skills (Bertollo & Yerys, 2019; Peterson et al., 2015; Udhnani et al., 2020). The relationship between EFs and AB has been observed in children with ASD across varying levels of intellectual functioning (Bertollo & Yerys, 2019; Gardiner & Iarocci, 2018; Pugliese et al., 2016). A longitudinal study by Pugliese et al. (2016) further supported that prior executive level predicted later AB in daily living skills and socialization, even after controlling for age and IQ. For children with ASD and an IQ ≤ 70, all domains of AB have displayed significant associations with executive deficits, such as working memory, initiation of action, self-monitoring, and organizational skills. Notably, working memory seems to play a crucial role in communication abilities and practical skills development (Pugliese et al., 2016; Powell et al., 2022; Hedvall et al., 2013), while behavioral regulation components like inhibition and cognitive flexibility play a significant role in social adaptation (Bertollo & Yerys, 2019).

Beyond IQ and EFs, other factors such as functional language abilities, ASD symptom severity, socioeconomic status, gender, and age influence AB in children with ASD. Delayed speech emergence is associated with more severe functional difficulties later in life (Mayo et al., 2013). Verbal children with ASD tend to have better adaptive skills than nonverbal children, who often show reduced abilities across various AB domains (Yang et al., 2016). Current research on the association between autism symptom severity and AB does not present consistent results. Several studies reported negligible to no associations between symptoms severity and overall AB (Hodge et al., 2021; Pathak et al., 2019; Wang et al., 2023) others identified a connection between higher symptom severity, particularly in social communication and repetitive behaviors, and lower overall adaptive functioning (Casula et al., 2024; Davico et al., 2022; Operto et al., 2021; Tillmann et al., 2019). Studies about the relationship between socioeconomic status and AB in individuals with ASD are also inconclusive. Gender, on the other hand, showed no significant association with overall AB (Pathak et al., 2019).

### Study Objectives

In the preschool period, the difficulties in AB, EFs, as well as in the intellectual functioning of children with ASD, are partly mapped. However, fewer studies have focused on this early developmental stage compared to school-aged children. A paucity of work looks more specifically at the cognitive ability structure captured by intelligence tests in preschool children with ASD. In older children, the profile of cognitive abilities shows unevenness, and some specificities are related to weaknesses and strengths. Therefore, we consider it essential not only to follow the overall intellectual functioning of children with ASD but also the broad cognitive abilities. The structure of EFs is less evident in the preschool period; however, the role of the three components mentioned earlier (inhibition, working memory, and shifting) is generally considered essential. Children with ASD also have an uneven profile of EFs, with various impairments. The overlap of cognitive abilities and EFs is more significant in this preschool period than later, so we assume the necessity to include both simultaneously if we consider how these abilities are associated with AB. Both cognitive abilities and EFs are crucial for independent functioning, achieving developmental milestones, and long-term adaptive outcomes.

This study aims to compare broad cognitive abilities, such as visuospatial abilities and reasoning and EFs, as high cognitive abilities through a model of three core components: inhibition, working memory, cognitive flexibility, as well as AB in preschool-aged children with ASD and TD children. Additionally, the study aims to analyze how cognitive abilities and EFs contribute to variability in domain-specific and global components of adaptive functioning in non- or minimally verbal preschool‐aged children with ASD. We considered examining these relationships important for contextualizing the results within typical development.

## Methods

### Participants

The total sample (*N* = 132) consisted of a clinical group of non- or minimally verbal children with ASD and a control group of TD children.

*ASD group*. The clinical group consisted of 53 children with ASD. Inclusion criteria required a confirmed diagnosis of ASD based on standardized assessments, specifically the Autism Diagnostic Observation Schedule, Second Edition (ADOS-2; Lord et al., 2012), and the Autism Diagnostic Interview-Revised (ADI-R; Rutter et al., 2003) with the diagnosis further validated by an independent clinician. Participants were also required to be between 3 and 6 years of age. The clinical sample consisted of 71% boys (*n* = 38) aged 3 to 6 years (M = 4.3; SD = 0.85). Of the sample, 8% of subjects presented with low severity of ASD symptoms, 45% with moderate symptoms, and 47% presented with severe symptoms (ADOS-2 calibrated severity scores). The IQ of the ASD group ranged from 48 to 128 (M = 91; SD = 24). 24.5% (*n* = 13) had intellect < 70 IQ. Based on parent reports and direct child observation, functional language impairments were evaluated according to ICD-11 (World Health Organization, 2019). This classification does not determine the severity of language impairments in different language domains, only the degree of impairment in functional language. The degrees are as follows: (1) “non-verbal” child - does not speak or can use a few words or expressions instead of words; (2) “limited verbal” child - uses simple three-word phrases, often containing a verb; (3) “fluent verbal” child - uses fluent, non-echoic speech. They speak in mostly correct sentences, demonstrate flexible language use, and may occasionally make grammatical errors. Our sample comprised 55% of non-verbal children and 45% of children with limited verbal expression. No child was verbally fluent.

#### *TD group*

The control group consisted of 79 typically developing children. The inclusion criteria were age between 3 and 6 years and achieving typical developmental milestones, including language milestones, as reported by parents. The exclusion criteria were the presence of any neurodevelopmental, neurological, sensory, or psychiatric disorders. The TD group included 52% of boys (*n* = 41). The age range was between 3 and 6 years (M = 4.9; SD = 0.91). The IQ of the TD group ranged from 85 to 144 (M = 114; SD = 12).

### Measures

#### Intellect (SON-R 2½ -7)

SON-R 2½-7 (Snijders-Oomen Nonverbal Intelligence Test; Tellegen et al., 2009) is a nonverbal measurement of intelligence in children aged 2–7 years. The test is not grounded in any specific theory of intelligence; however, from the perspective of the Cattell-Horn-Carroll (CHC) theory of intelligence, the SON-R subtests primarily assess fluid intelligence (Gf) and visual processing abilities (Gv) (Schneider & McGrew, 2012). The test comprises six subtests, which produce two composite domain scores: Performance and Reasoning. The *Performance* scale assesses spatial orientation, planning, and concrete thinking, while the *Reasoning* scale evaluates abstract and logical thinking. For the Slovak test version, Cronbach’s α was 0.861 for the *Performance* scale, 0.796 for the *Reasoning* scale, and 0.898 for the total score (Dočkal, 2012). Our study administered the test in full, including all subtests.

### Executive Functioning (BRIEF-P)

The level of executive functions was evaluated by the rating scale BRIEF-P (Behavior Rating Inventory of Executive Functioning–Preschool Version; Gioia et al., 2003) developed to evaluate everyday behaviors associated with executive functioning in children aged 2 to 5 years. The questionnaire consists of 63 items, five clinical scales (Inhibition, Shifting, Emotional control, Working memory, Planning/Organization), three clinical composite indexes, and a Global Executive Composite. The completion takes approximately 10–15 min, and a three-point frequency scale (1 – never, 2 – sometimes, 3 – often) is used to evaluate how often the problematic behavior occurred during the past 6 months of the child’s behavior (Gioia et al., 2003). Higher scores reflect executive difficulties. The parents filled out the questionnaire. According to the authors, the internal consistency of the BRIEF-P scales (version for parents) is high; Cronbach’s α was at the level of 0.80 to 0.90 (Gioia et al., 2003). We examined executive functions through a model of three core components: inhibition, working memory, and cognitive flexibility (Diamond, 2013; Zelazo & Carlson, 2012). For this purpose, we used *Inhibition*, *Shifting*, and *Working memory* scales.

### Adaptive Behavior (VABS-3)

VABS-3 (The Vineland Adaptive Behavior Scales – Third Edition; Sparrow et al., 2016) assesses AB and includes three domains: *Communication*, *Daily Living Skills*, and *Socialization*, and the composite score ABC, which reflects overall adaptive functioning. The scale captures how often the observed behavior occurs without help or needing a reminder. Responses are expressed on a three-point scale (0 - never, 1 - sometimes, 2 - usually or often) or dichotomously (2 - yes, 0 - no). The child’s parents filled out the questionnaire. We used the Slovak research version with Cronbach’s α for scale, ranging from 0.92 to 0.98.

### Procedures

This study was performed in line with the principles of the Declaration of Helsinki and the approval was granted by the Ethics Committee of University Hospital Bratislava Old Town and the Faculty of Medicine of the Comenius University in Bratislava (Approval No. 78/2021). Data were collected from 01/2022 to 03/2023 at the Academic Research Center for Autism, Faculty of Medicine, and the Faculty of Arts at Comenius University in Bratislava. The clinical sample was based on an examination of children with at-risk neurodevelopmental disorders who participated in multiple assessment sessions. Written informed consent was obtained from all parents or legal guardians prior to participation. Due to the young age of the participants, formal assent from the children was not required, but participation was voluntary and child-friendly procedures were used to ensure their comfort. The final ASD sample included in statistical analyses resulted from several steps: (1) 85 children met the diagnostic criteria for ASD (based on ADOS-2, ADI-R, independent clinician confirmation of diagnosis); (2) intellectual abilities (SON-R 2½ -7) could be assessed in 61 children who met the criteria of ASD; 24 children were excluded due to poor cooperation, difficulty understanding instructions, or low behavioral regulation, and (3) the final sample was represented by 53 children who completed intellect testing, and who had complete parent-rated data on AB (VABS-3) and EFs (BRIEF-P); these data were missing in 8 participants, therefore they were excluded. The protocol of performance-based tests also included EFs tests, including the experimental online (via tablet) inhibition task Go/No-Go test and a working memory span task based on the “Picture Memory” (WPPSI-IV; Wechsler, 2012) task. Performance-based tasks in the ASD group were completed by 36 children from the final sample (*n* = 53). 32% of performance-based EFs data was missing. The TD group was recruited through website announcements and based on parental reports regarding the child’s development. 83 children met the inclusion criteria for the TD group. Children participated in a single testing session (70–90 min) that involved performance assessments using the SON-R 2½ -7 and performance-based EFs tasks. During testing, two children were excluded from the TD group due to challenging behavior that prevented the completion of assessments. Parent reports were missing in 2,5% (*n* = 2) of participants who mastered intellect testing. Incomplete performance-based assessment of EFs was in 10% of participants who met the inclusive criteria for the TD group. However, due to many incomplete protocols among children with ASD, primarily attributed to difficulty in understanding instructions, test refusal, or distractions during testing (e.g., sensory interests), these performance-based measures were excluded from the analyses. Consequently, the parent-reported BRIEF-P was used to evaluate executive functioning.

### Statistical Analyses

Statistical analysis was conducted using the R 4.3.2 software. VABS-3, BRIEF-P, and SON-R 2½-7 data were transformed into weighted scores. Independent t-tests were used to compare groups and analyze gender differences. Effect sizes were calculated and interpreted according to Cohen’s recommendations: small (d = 0.2), medium (d = 0.5), and large (d = 0.8) (Cohen, 1992). Pearson correlation coefficients were calculated to explore the association between cognitive predictors and AB outcomes. In the main part of the analysis, four multiple linear regression models were computed for ASD and TD groups. To determine the association between AB and cognitive abilities, each multiple regression model (Enter method) used the VABS-3 domain score as the dependent variable and domains of intellect (*Reasoning*, *Performance*) and executive functions (*Inhibition*, *Working memory*, *Shifting*) as predictors. The assumption checks showed that the data met the linearity and residual normality criteria for multiple linear regression. No significant gender differences were found in the measured variables (see Table S1 in Supplement). We calculated minimum effect sizes detectable at our sample size in a power analysis using G*Power 3.1.9.7. With alpha set to 0.05, the analysis indicated 80% power to detect medium to large effect size for the ASD group and medium effect size for our TD group.

## Results

### Descriptive Statistics

Descriptive data of all studied variables in the ASD and TD groups are displayed in Table 1. The distribution of scores showed expected ranges and differences between groups with medium to large effects. Children with ASD scored significantly worse in all measures than TD children (Table 1). TD children performed at average levels in cognitive abilities, displaying low executive difficulties and average AB scores typical for their age. In the ASD group, the clinically significant range of high scores (T-scores ≥ 65) of EFs was observed in 58% (vs. 16.5% in the TD group) in *Inhibition*, 35% in *Shifting* (vs. 11% in the TD group) and 69% in *Working memory* (vs. 20% TD group), respectively.

**Table 1:** Descriptive data for all model variables

		ASD (*n* = 53)				TD (*n* = 79)				t-test^a^			95% CI for Cohen’s d	
		*M*	*SD*	*Min*	*Max*	*M*	*SD*	*Min*	*Max*		*t*	*d*	Lower	Upper
*Adaptive behavior* (VABS-3)	COM	72.3	17.68	36	107	97.8	11.00	70	122		10.26	1.8	1.37	2.27
	DLS	79.9	13.51	54	114	98.3	10.86	76	132		8.68	1.5	1.11	1.96
	SOC	73.4	13.19	50	101	96.9	9.29	76	120		12.2	2.1	1.65	2.62
	ABC	75.0	11.95	53	107	96.5	10.92	76	127		10.63	1.9	1.43	2.35
*Cognitive domain* (SON-R 2½-7)	PS IQ	92.6	19.94	58	125	111.7	11.57	83	145		7.2	1.2	0.844	1.64
	RS IQ	92.2	26.07	40	136	113.8	13.77	77	146		6.24	1.1	0.717	1.49
*Executive functions* (BRIEF-P)	INH	67.9	13.41	40	92	54.9	11.26	37	91		7.34	-1.1	-1.44	-0.67
	SHI	59.2	13.42	37	89	50.5	10.20	38	89		-5.96	-0.7	-1.08	-0.35
	WM	70.8	15.88	40	99	54.7	11.84	36	96		-4.06	-1.2	-1.56	-0.77

Note. All scores are standardized, VABS-3 & SON-R (*M* = 100, *SD* = 15), BRIEF-P (*M* = 50, *SD* = 10); ASD – autism spectrum disorder, TD – typically developing children, *M* – mean, *SD* – standard deviation, *Min* – minimum, *Max* – maximum, *t*-test statistic, *d* – effect size; *CI* – Confidence Interval; *COM* – Communication, *DLS* – Daily Living Skills, *SOC* – Socialization, *ABC* – composite score; *PS IQ* – Performance, *RS IQ* – Reasoning; *INH* – Inbibition, *SHI* – Shifting, *WM* -Working memory

^*a*^ degrees of freedom = 130; all t-tests were significant at *p* <.001

Correlations of AB domains with scales measuring intellect and EFs for both groups are displayed in Supplement Tables 2 and 3. In the ASD group, all AB domains and the composite score were significantly correlated with both cognitive domains (from 0.29 to 0.69) and with two executive domains – *Inhibition* (form − 0.40 and − 0.49) and *Working memory* (from − 0.54 to − 0.66) *-* ranging from low to high degree. *Shifting* was unrelated to the AB domains or composite ABC score. In the TD group, AB did not correlate with cognitive ability or EFs.

Multiple linear regressions were used to test if cognitive domains and executive difficulties significantly predicted AB domains. In the ASD group, the *Working memory* scale was the strongest predictor of the composite AB score (ABC) while also significantly predicting all three AB domains (Table 2). *Communication* domain was also significantly predicted by *Reasoning*. In the TD group, no significant predictors were observed in the models (Table 3).

**Table 2:** Regression models of AB domains for children with ASD

Predictor	COM	DLS	SOC	ABC
(Intercept)	68.44***	99.29***	95.46***	91.35***
Performance scale (SON-R)	0.14	0.06	-0.05	0.03
Reasoning scale (SON-R)	0.32***	0.04	0.175	0.12
Inhibition (BRIEF-P)	-0.20	0.02	0.047	0.06
Shifting (BRIEF-P)	-0.01	-0.01	-0.161	-0.04
Working Memory (BRIEF-P)	-0.34*	-0.41*	-0.39*	-0.45**
*Model adj. R* ^*2*^	0.681***	0.237**	0.314 ***	0.463***

Note. *n* – 53; VABS-3: *COM* – Communication, *DLS* – Daily Living Skills, *SOC* – Socialization, *ABC* – composite score; * *p* <.05, ** *p* <.01, *** *p* <.001

**Table 3:** Regression models of AB domains for TD children

Predictor	COM	DLS	SOC	ABC
(Intercept)	106.08***	103.35***	120.89***	111.40***
Performance scale (SON-R)	-0.01	-0.09	-0.11	-0.08
Reasoning scale (SON-R)	0.07	0.11	0.002	0.07
Inhibition (BRIEF-P)	-0.06	0.19	-0.06	0.02
Shifting (BRIEF-P)	-0.10	-0.04	-0.11	-0.10
Working Memory (BRIEF-P)	-0.12	-0.28	-0.07	-0.17
*Model adj. R* ^*2*^	− 0.002	− 0.003	− 0.007	− 0.012

Note. *n* – 79; VABS-3: *COM* – Communication, *DLS* – Daily Living Skills, *SOC* – Socialization, *ABC* – composite score; *** *p* <.001

## Discussion

This study compared cognitive abilities, EFs, and AB in non- or minimally verbal children with ASD and a control group of TD children. When comparing the ASD and TD groups, significant differences were observed in all independent and dependent variables, characterized by large effect sizes, except for *Shifting*, where we observed a medium effect.

Children with ASD exhibited more executive difficulties across all domains: *Inhibition*, *Shifting*, and *Working Memory*. The greatest difference was in *Working memory*, where children with ASD scored on average 1.5 SD of T-scores higher than TD children, and 69% of ASD children had clinically significantly elevated scores indicating more severe impairment. These findings align with a recent study on a larger cohort of preschoolers with ASD (Terroux et al., 2024). The difference between groups in *Shifting* was significant, with a medium effect size, but compared to other EFs, it represented the smallest effect. Prior studies on school-aged children with ASD have consistently reported that *Shifting* shows the largest difference on the BRIEF/BRIEF2 scale compared to TD children (Gioia et al., 2015; Granader et al., 2014; van den Bergh et al., 2014). This difference is probably related to natural developmental changes in cognitive flexibility; it is only at school age that the child’s cognitive flexibility skills increase sharply, in parallel with the increasing demands of the environment on the child (Garon et al., 2008). These findings suggest that *Shifting* difficulties may not be as clinically significant in preschool-aged children with ASD compared to TD children as they are at later ages. Starting school introduces new demands, and parents may find rigid behavior more disruptive as it manifests across different environments, making problems in this area more noticeable in older children. Additionally, parents of children with ASD may have adapted to their EFs difficulties, potentially underestimating their severity and not reporting them as accurately. Similar results were observed in other studies with preschoolers when assessing executive difficulties through parental evaluations (Smithson et al., 2013; Terroux et al., 2024).

Significant group differences in cognitive abilities were found between children with ASD and TD controls, with the TD group overperforming the ASD group in *Reasoning* and *Performance* scales. The ASD group showed greater variability in cognitive performance, with intellectual abilities ranging from − 4 SD to + 2.5 SD above the mean. In our sample, 25% of children had an IQ < 70, which is a common prevalence in children with ASD (Maenner, 2023).

Significant differences in all domains of AB were observed between TD and ASD groups. The largest effect was in the *Socialization* domain, and the smallest, though still large, was in *Daily Living Skills*. The ASD group had similar levels in the *Communication* and *Socialization* domains, contrary to previous research suggesting more severe *Socialization* impairment (Kanne et al., 2011; Saulnier et al., 2022). These results probably reflect the characteristics of our sample of ASD children with limited expressive language abilities, which may have affected the level of the *Communication* domain in particular.

The second aim of this study was to analyze cognitive ability and EFs as predictors of AB domains in non- or minimally verbal preschool children with ASD and TD children. Correlation analysis of predictors and AB domains yielded different results in the ASD and TD groups. In the ASD group, *Reasoning*,* Performance* scales, *Working Memory*, and *Inhibition* had associations with AB domains, ranging from weak to strong. *Shifting* had no relationship with these domains, unlike previous studies that reported a weak negative correlation (Gardiner & Iarocci, 2018; Terroux et al., 2024). In the TD group, both executive difficulties and cognitive domains exhibited no significant correlations with AB domains. The lower the intellectual functioning, the more AB corresponds to its level in the general population. Conversely, in average and above-average intellect, the relationship between intellectual functioning and AB is weak or non-existent (Alexander & Reynolds, 2020). The association between AB and intellect is higher at younger ages. Children in our sample had average to above-average intellect.

Before analyzing the models, we conducted gender analyses on the variables within both groups, revealing non-significant differences consistent with findings from prior studies (Powell et al., 2022). Similarly, the autistic symptoms were unrelated to any domain, so we did not include them in the regression models for the ASD group.

Our results show that cognitive domains and executive difficulties account for up to 68% of the variance in the *Communication* domain in the ASD group. Reasoning and difficulties in *Working Memory* are significant predictors. According to prior research, intellectual abilities appear closely linked to conceptual skills (Alexander & Reynolds, 2020; Sabat et al., 2019). The *Communication* domain in the VABS-3 is a component of the conceptual domain, as defined by ICD-11 (WHO, 2019). It includes academic skills like reading and writing, which are relevant for preschool literacy predictors such as symbol recognition and name writing. Items in this domain are linked to EFs, mainly working memory (e.g., following instructions). The *Reasoning* domain involves fluid intelligence skills, including induction and categorization, with some subtests requiring verbal reasoning despite nonverbal administration (Dočkal, 2012; Tellegen et al., 2009).

Next, the cognitive domains and executive functions explained 23% of the variance in the *Daily Living Skills* domain in the ASD group, with *Working memory* being the only significant predictor. *Daily Living Skills* include self-care tasks like dressing, eating, and tidying up. This domain is generally less impaired in children with ASD than other domains (Kanne et al., 2011; Saulnier et al., 2022), and our sample presented the highest average scores in this area. These skills can be effectively trained in children with intellectual disabilities or developmental difficulties, provided no significant motor impairments (Adjorlu et al., 2017; Lamash et al., 2017; Matsumura et al., 2022).

Finally, cognitive domains and executive functions explain 31% of the variance in the *Socialization* domain in the ASD group, with *Working memory* emerging as the only significant predictor. This domain evaluates behaviors essential for interpersonal interactions, such as participating in joint activities, responding appropriately in social contexts, and forming friendships. Previous research on socialization and EFs in ASD has yielded mixed results. Some studies have found associations between shifting in school-aged children (Bertollo et al., 2020; Pugliese et al., 2015) and working memory (Freeman et al., 2017; Leung et al., 2015). Emotional control has also been linked to socialization in children with ASD without co-occurring developmental delays (Braverman et al., 2024). However, these studies primarily focused on verbally competent children, while our sample is mainly nonverbal or minimally verbal. The number of nonverbal children in our sample may affect the relationship between working memory and social skills, particularly given its potential connection to verbal abilities in younger age groups (Edmunds et al., 2022).

The composite *ABC* score was included in the analysis to reflect the overall AB. The cognitive domain and executive difficulties explained 46% of the variability in *ABC* score in the ASD group. Also, the *Working Memory* deficits exhibited a significant predictive power, but cognitive abilities were not significant predictors of the *ABC* score. Hedvall et al. (2013) analyzed cognitive abilities as predictors of overall AB in preschool children with ASD. Verbal ability and information processing speed were identified as significant predictors; we did not observe these in our study. On the other hand, the performance domain associated with visuospatial abilities was not a significant predictor of AB, which is consistent with our results. The result of this analysis may also have been influenced by the fact that in our sample, most children did not have an intellectual disability. This is consistent with prior studies highlighting a pronounced discrepancy between cognitive abilities and AB in this population (Alvares et al., 2020; Pathak et al., 2019). These results suggest that EFs, particularly *Working Memory*, hold greater predictive significance for AB quality in preschoolers than visuospatial abilities and reasoning.

Neither *Inhibition* nor *Shifting* emerged as significant predictors of AB in the ASD group. Several studies have found that *Shifting* scores are significantly elevated compared to other executive components in individuals with ASD (Christoforou et al., 2023; Gardiner & Iarocci, 2018; Gioia et al., 2015; Granader et al., 2014), likely due to core ASD traits of rigidity and insistence on sameness, which impede adaptation to new situations.

Analysis of cognitive ability and EFs as predictors for AB in the TD group did not support any of the models. A weak or non-existent relationship between AB and cognitive domains is expected in children developing without neurodevelopmental impairments, particularly when IQ is above 100 (Alexander & Reynolds, 2020). In our TD sample, the average IQ scores in both cognitive domains were above 100, with intellectual performance skewed toward the higher end of the average range. Although some studies have found a significant relationship between behavioral regulation, particularly impulse inhibition, and socialization in school-aged TD children (Demkaninová et al., 2022), this effect is less evident in preschoolers.

Assessing EFs using rating scales yields slightly different results than performance tests (Faridi et al., 2015). Our study accounted for this by multi-source assessment. However, during data collection, we encountered issues with the limits in the ASD group in terms of self-regulation and ability to understand instructions of tablet-based tasks for inhibition and memory, leading to data exclusion for younger children. In the TD group, significantly more children could complete the protocol. Overall, this raises questions about the validity of the evaluation of EFs, especially at a young age in children with ASD.

These aspects make it challenging to apply a three-component executive model. In the preschool period, some research suggests that it is more meaningful to examine executive functions as a common factor (Best et al., 2009; Friedman et al., 2008) rather than individual components that are still undergoing development. On the other hand, it is important to consider the clinical setting of the study and previous findings, which have shown that the EF profile of children with ASD is uneven, with specific peaks that may significantly influence relationships with AB domains. One possibility in the future is to incorporate other aspects of EFs, such as emotional regulation, planning, and organizing (Gioia et al., 2003), which may also play an essential role in child functioning.

In the ASD group, autistic symptom severity showed no significant correlations with either the independent or dependent variables in our study, consistent with findings by Smithson et al. (2013) and Hodge et al. (2021), who reported that symptom severity did not predict variability in adaptive functioning. Conversely, Terroux et al. (2024) found a positive association between executive difficulties and higher symptom severity, which may be due to methodological differences. Our study utilized a performance-based assessment, while Terroux et al. (2024) evaluated autism symptom severity through parent-reported responses. This discrepancy suggests that parent reports may be biased and not fully capture an individual’s abilities compared to performance-based assessments.

### Strengths, Limitations, and Avenues for Future Research

Our study has several limitations related to the research sample and the cognitive and executive assessment tools. Evaluating cognitive functions in children with ASD poses challenges for clinicians and researchers (Courchesne et al., 2019; Klinger et al., 2018). In our study, up to 28% of children who met inclusion criteria could not undergo cognitive assessment using the nonverbal intelligence test SON-R 2½-7. Substantial challenges in administering the performance test in the ASD group included difficulties in comprehending instructions, the ability to concentrate, and a certain level of behavior regulation. Although SON-R 2½-7 can be administered non-verbally, it requires some ability to understand the social situation in which the child is cooperating with the administrator. Therefore, our research sample likely included children with better self-regulation and social competence. This bias is probably unavoidable in all research studies with younger children with ASD. Similar challenges in cognitive assessment were observed by Studer et al. (2017), who also used SON-R 2½-7. In their study of preschool children with ASD, only 26% could initially complete the testing; however, after one year of intervention, this increased to 86%, and after two years, to 100%. These results highlight the importance of early intervention, not only for improving child outcomes (Eikeseth et al., 2012; Estes et al., 2015a; Smith et al., 2021) but also allows for more valid information about the cognitive abilities of children with ASD. On the other side, our sample consisted of children with ASD who were not verbally fluent or non-verbal, which significantly complicates objective assessment and increases the risk of underestimating cognitive abilities, as demonstrated in previous studies (Courchesne et al., 2015, 2019). In contrast, the TD group consisted of verbally fluent children. This discrepancy results in a confounding factor, complicating the differentiation between the effects of autism and those of language ability. Therefore, limitations associated with the SON-R 2½-7 must be considered alongside the differences in language and cognitive profiles between the ASD and TD groups. The absence of children with lower verbal and cognitive abilities in the TD sample may have further contributed to group-level differences and limited the generalizability of our findings.

The absence of verbally fluent children in our sample does not align with their representation in other studies. This fact is related to the diagnostic options in our country, where verbally fluent children are often underdiagnosed due to insufficiently sensitive screening for those without significant language impairments.

The tool for assessing cognitive ability, the intelligence test SON-R 2½-7, is limited in scope. It does not capture language, memory skills, or information processing speed, which are significant predictors of AB. Processing speed is crucial in assessing young children due to its close relationship with other cognitive abilities and learning. Developmental research emphasizes the dynamic interplay between processing speed, working memory, and reasoning (Hedvall et al., 2013).

The choice of tool was limited by availability in our local context. Without control of verbal abilities, particularly in the *Communication* domain, but also in *Socialization*, we cannot definitively determine to what extent the observed relationships are a result of the presence of ASD or the children’s level of language competencies, as described in some studies (Bennett et al., 2014; Miranda et al., 2023). In the future, it will be necessary to consider these aspects.

The final sample size may not be sufficient to generalize the findings to the broader population of children with ASD. The ASD group also included participants with IQ lower than 70, and this level of intellect in children with ASD is differently associated with AB (Panerai et al., 2014). Intellectual disability could interact with other variables (e.g., executive function, age, severity of ASD symptoms), complicating the interpretation of results and potentially covering up significant relationships.

Future analyses should take into account other potential confounding variables, such as the presence of comorbid conditions (e.g., ADHD, anxiety), medication use, or the type and intensity of interventions received by children with ASD. These factors could influence both executive functions and AB.

## Conclusion

The study supported previous findings on cognitive abilities, EFs, and AB quality in non- or minimally verbal preschool-age children with ASD. It also contributed to understanding the relationship between cognitive abilities, EFs, and AB. Notably, working memory emerged as a key factor, predicting AB across key domains - Communication, Socialization, and Daily Living Skills—highlighting its importance for early intervention. While cognitive abilities, such as fluid reasoning, also contributed to communication skills, the absence of significant relationships between AB, cognitive abilities, and EFs in TD children supports the unique developmental specifics of children with ASD. The study highlights challenges in cognitive assessment for nonverbal or minimally verbal preschool children with ASD, pointing to a need for more inclusive tools that better accommodate varying self-regulation and communication abilities.

## Electronic Supplementary Material

Below is the link to the electronic supplementary material.

Supplementary Material 1
