# Supplementary material for: Cognitive Abilities and Executive Functions as Predictors of Adaptive Behavior in Preschoolers with Autism Spectrum Disorder and Typically Developing Children: A Comparative Study
Source: Res Child Adolesc Psychopathol. 2025 Jun 18;53(10):1525–38. doi: 10.1007/s10802-025-01341-x (PMC12521314; doi:10.1007/s10802-025-01341-x)
Supplement: Supplementary file 1 — Supplementary Material 1 [file 10802_2025_1341_MOESM1_ESM.docx]

**Supplemental Tables**

| **Table S1** Gender difference tests for the studied variables | | | | | | | | | | | |  |
| --- | --- | --- | --- | --- | --- | --- | --- | --- | --- | --- | --- | --- |
|  | | | | | | | | 95% *CI* for Cohen’s d | | | |  |
|  | | *t* | | *p* | | *d* | | Lower | | | Upper |  |
| PS IQ – Performance |  | 0.539 |  |  | 0.592 |  | 0.164 |  |  | -0.435 | 0.762 |  |
| RS IQ – Reasoning |  | 0.471 |  |  | 0.639 |  | 0.144 |  |  | -0.455 | 0.741 |  |
| Inhibition |  | 1.070 |  |  | 0.290 |  | 0.326 |  |  | -0.276 | 0.926 |  |
| Shifting |  | -0.305 |  |  | 0.762 |  | -0.093 |  |  | -0.690 | 0.505 |  |
| Working memory |  | 0.146 |  |  | 0.884 |  | 0.045 |  |  | -0.553 | 0.642 |  |
| COM |  | 0.323 |  |  | 0.748 |  | 0.099 |  |  | -0.500 | 0.696 |  |
| DLS |  | -0.458 |  |  | 0.649 |  | -0.140 |  |  | -0.737 | 0.459 |  |
| SOC |  | -0.636 |  |  | 0.528 |  | -0.194 |  |  | -0.792 | 0.406 |  |
| ABC |  | -0.177 |  |  | 0.860 |  | -0.054 |  |  | -0.651 | 0.544 |  |
| *Note.* -test statistic, *d* – effect size; *CI* – Confidence Interval; *COM* – Communication, *DLS* – Daily Living Skills, *SOC* – Socialization, *ABC* – composite score. | | | | | | | | | | | |  |

| **Table S2**  *Pearson’s Correlations of the studied variables in ASD group* | | | | | | | | | | | | | | | | | | | | | | | | | | | | |  |  |  |
| --- | --- | --- | --- | --- | --- | --- | --- | --- | --- | --- | --- | --- | --- | --- | --- | --- | --- | --- | --- | --- | --- | --- | --- | --- | --- | --- | --- | --- | --- | --- | --- |
|  | **1** | | | | **2** | | | **3** | | | **4** | | | **5** | | | **6** | | | **7** | | | **8** | | | **9** | | |  |  |  |
| 1. PS IQ – Performance |  |  | — |  | |  |  | |  |  | |  |  | |  |  | |  |  | |  |  | |  |  | |  |  | |  |  |
| 2. RS IQ – Reasoning |  |  | 0.769 | *** | | — |  | |  |  | |  |  | |  |  | |  |  | |  |  | |  |  | |  |  | |  |  |
| 3. Inhibition |  |  | -0.252 |  | | -0.124 |  | | — |  | |  |  | |  |  | |  |  | |  |  | |  |  | |  |  | |  |  |
| 4. Shifting |  |  | 0.093 |  | | 0.230 |  | | 0.427 | ** | | — |  | |  |  | |  |  | |  |  | |  |  | |  |  | |  |  |
| 5. Working memory |  |  | -0.434 | ** | | -0.311 | * | | 0.787 | *** | | 0.314 | * | | — |  | |  |  | |  |  | |  |  | |  |  | |  |  |
| 6. COM |  |  | 0.689 | *** | | 0.704 | *** | | -0.493 | *** | | -0.046 |  | | -0.642 | *** | | — |  | |  |  | |  |  | |  |  | |  |  |
| 7. DLS |  |  | 0.354 | ** | | 0.294 | * | | -0.401 | ** | | -0.125 |  | | -0.538 | *** | | 0.665 | *** | | — |  | |  |  | |  |  | |  |  |
| 8. SOC |  |  | 0.366 | ** | | 0.389 | ** | | -0.412 | ** | | -0.217 |  | | -0.554 | *** | | 0.678 | *** | | 0.840 | *** | | — |  | |  |  | |  |  |
| 9. ABC |  |  | 0.489 | *** | | 0.464 | *** | | -0.468 | *** | | -0.142 |  | | -0.661 | *** | | 0.856 | *** | | 0.919 | *** | | 0.880 | *** | | — |  | |  |  |
|  | | | | | | | | | | | | | | | | | | | | | | | | | | | | |  |  |  |
| *Note*. *n* = 53; *COM* – Communication, *DLS* – Daily Living Skills, *SOC* – Socialization, *ABC* – composite score of adaptive behavior.  * p < .05, ** p < .01, *** p < .001 | | | | | | | | | | | | | | | | | | | | | | | | | | | | |  |  |  |

| **Table S3**  *Pearson’s Correlations of the studied variables in the TD group* | | | | | | | | | | | | | | | | | | | | | | | | | | | | |  |  |  |
| --- | --- | --- | --- | --- | --- | --- | --- | --- | --- | --- | --- | --- | --- | --- | --- | --- | --- | --- | --- | --- | --- | --- | --- | --- | --- | --- | --- | --- | --- | --- | --- |
|  | **1** | | | | **2** | | | **3** | | | **4** | | | **5** | | | **6** | | | **7** | | | **8** | | | **9** | | |  |  |  |
| 1. PS IQ – Performance |  |  | — |  | |  |  | |  |  | |  |  | |  |  | |  |  | |  |  | |  |  | |  |  | |  |  |
| 2. RS IQ – Reasoning |  |  | 0.378 | *** | | — |  | |  |  | |  |  | |  |  | |  |  | |  |  | |  |  | |  |  | |  |  |
| 3. Inhibition |  |  | 0.053 |  | | -0.032 |  | | — |  | |  |  | |  |  | |  |  | |  |  | |  |  | |  |  | |  |  |
| 4. Shifting |  |  | -0.107 |  | | -0.006 |  | | 0.341 | ** | | — |  | |  |  | |  |  | |  |  | |  |  | |  |  | |  |  |
| 5. Working memory |  |  | -0.196 |  | | -0.056 |  | | 0.668 | *** | | 0.406 | *** | | — |  | |  |  | |  |  | |  |  | |  |  | |  |  |
| 6. COM |  |  | 0.054 |  | | 0.094 |  | | -0.183 |  | | -0.167 |  | | -0.213 |  | | — |  | |  |  | |  |  | |  |  | |  |  |
| 7. DLS |  |  | 0.026 |  | | 0.109 |  | | -0.028 |  | | -0.084 |  | | -0.174 |  | | 0.676 | *** | | — |  | |  |  | |  |  | |  |  |
| 8. SOC |  |  | -0.104 |  | | -0.039 |  | | -0.174 |  | | -0.164 |  | | -0.157 |  | | 0.613 | *** | | 0.579 | *** | | — |  | |  |  | |  |  |
| 9. ABC |  |  | -0.007 |  | | 0.060 |  | | -0.142 |  | | -0.151 |  | | -0.198 |  | | 0.876 | *** | | 0.848 | *** | | 0.805 | *** | | — |  | |  |  |
|  | | | | | | | | | | | | | | | | | | | | | | | | | | | | |  |  |  |
| *Note*. *n* = 53; *COM* – Communication, *DLS* – Daily Living Skills, *SOC* – Socialization, *ABC* – composite score of adaptive behavior.  * p < .05, ** p < .01, *** p < .001 | | | | | | | | | | | | | | | | | | | | | | | | | | | | |  |  |  |
